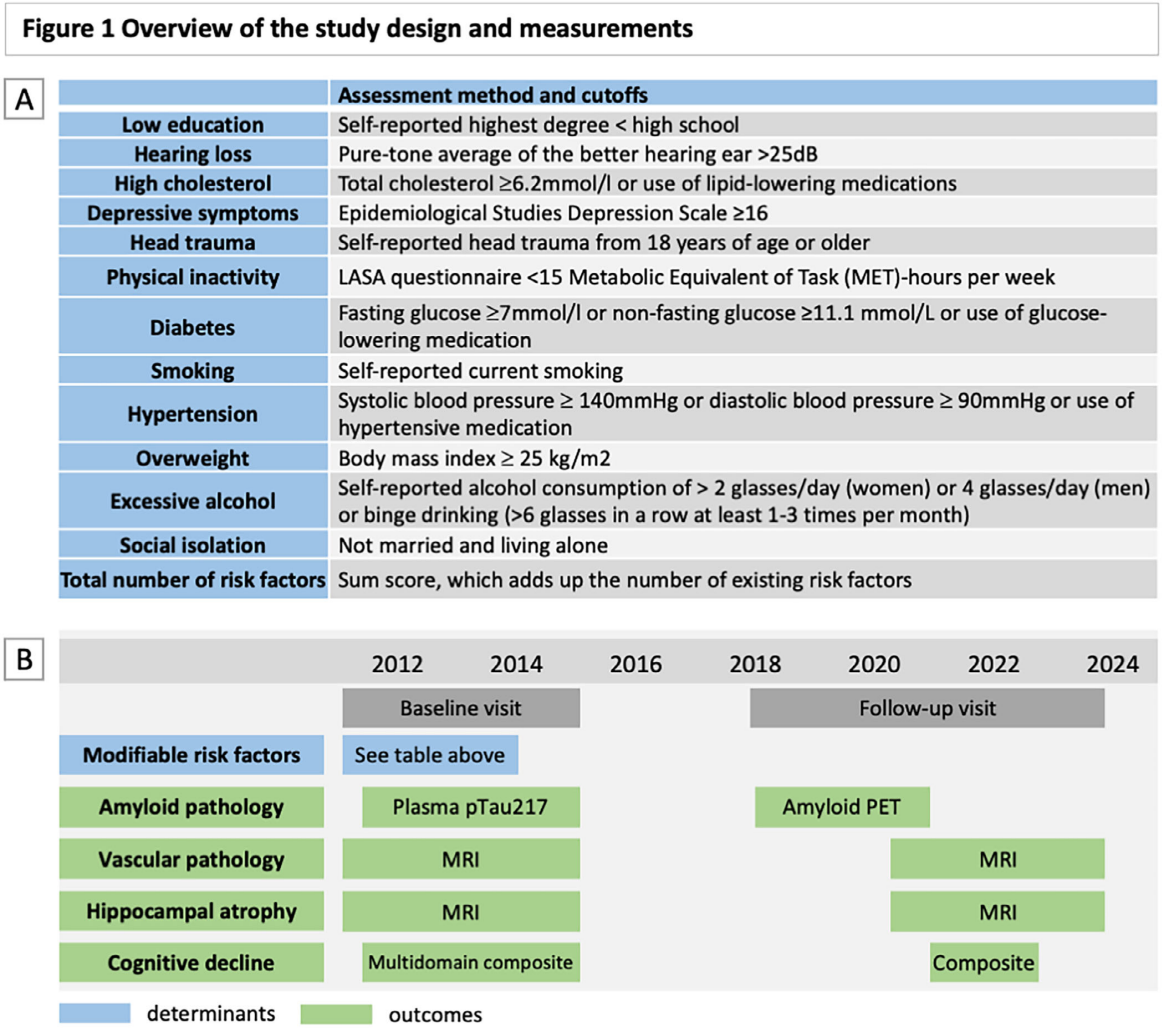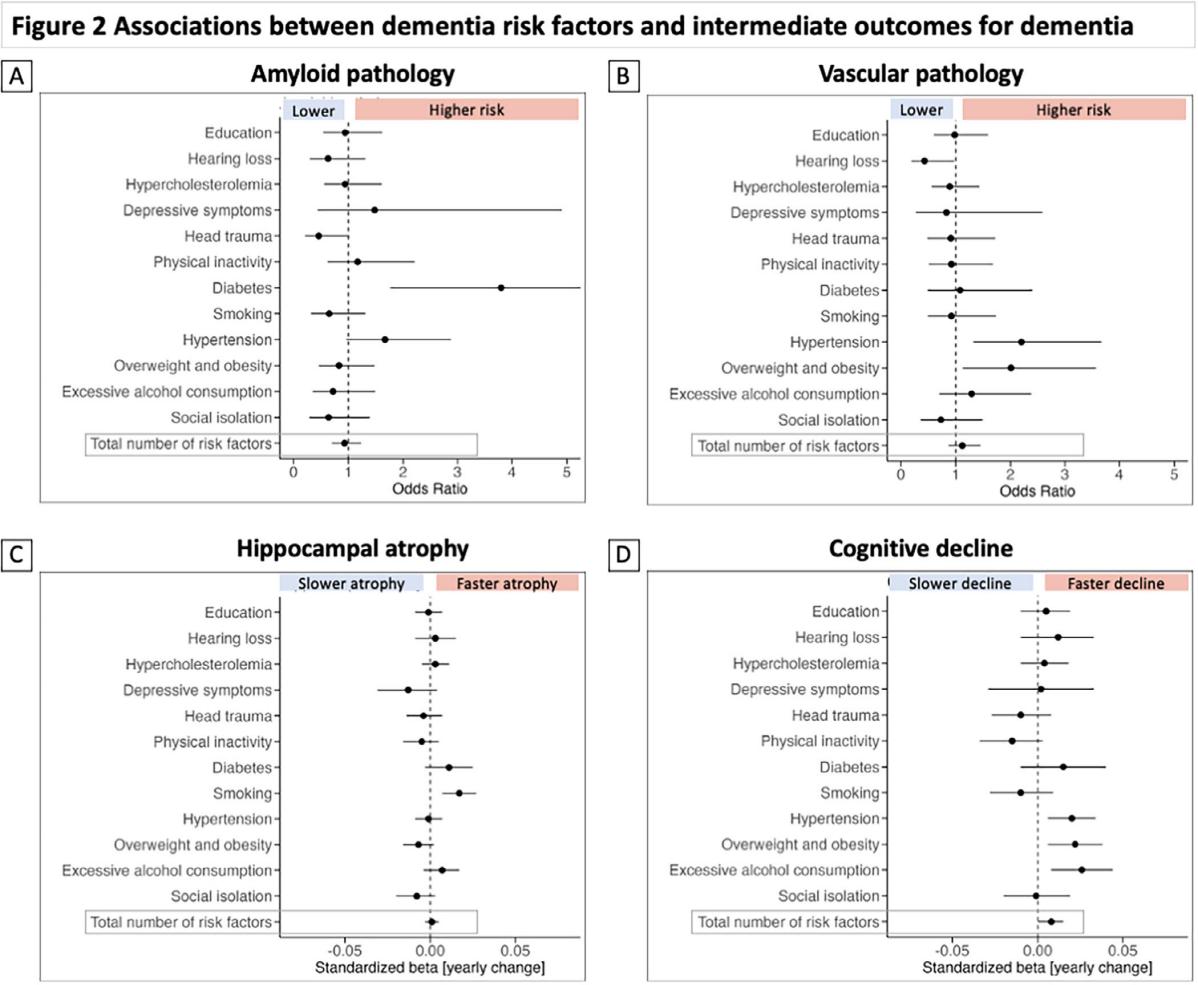# Modifiable dementia risk factors differentially relate to intermediate brain and cognitive outcomes in the population‐based Rotterdam Study

**DOI:** 10.1002/alz70860_098366

**Published:** 2025-12-23

**Authors:** Phuong Thuy Nguyen Ho, Anna van Houwelingen, Elisabeth J. Vinke, Jordi H.C. Boons, André Goedegebure, Miranda Schram, Arfan Ikram, Frank J. Wolters, Trudy Voortman, Meike W. Vernooij, Julia Neitzel

**Affiliations:** ^1^ Erasmus University Medical Center, Rotterdam, Netherlands; ^2^ Maastricht University Medical Centre, Maastricht, Netherlands; ^3^ Harvard T.H. Chan School of Public Health, Boston, MA, USA

## Abstract

**Background:**

The Lancet Commission lists 14 potentially modifiable risk factors that are jointly held responsible for a substantial number of dementia cases. To better understand the brain mechanisms through which these factors increase dementia risk, we investigated their associations with four intermediate dementia‐related outcomes: (1) amyloid pathology, (2) vascular pathology, (3) hippocampal atrophy, and (4) cognitive decline.

**Method:**

We included 635 dementia‐free participants from the prospective population‐based Rotterdam Study who had an amyloid PET scan. At baseline (2011‐2015), 12 modifiable risk factors were collected (Figure 1A). For outcome (1), we measured Simoa Alzpath pTau217 positivity (≥0.63pg/mL) at baseline and amyloid ^18^F‐florbetaben PET positivity on average seven years later (Figure 1B). For outcome (2), we determined vascular pathology, defined as small vessel disease or a cortical infarct, on baseline and on follow‐up MRI about nine years later. For outcome (3), bilateral hippocampal volume was extracted on the baseline and follow‐up MRIs. For outcome (4), we computed a multi‐domain cognitive composite score at baseline and follow‐up. We used logistic regressions to examine the association between modifiable risk factors and incident amyloid and vascular pathology, excluding participants with prevalent pathology at baseline. Mixed effects models were used to estimate associations between risk factors and hippocampal atrophy and cognitive decline over time. Analyses were adjusted for age, sex, APOE4, intracranial volume and education.

**Result:**

We found that diabetes and, to a lesser extent, hypertension were associated with a higher risk of amyloid PET positivity at follow‐up (Figure 2A). Hypertension and overweight were associated with a higher risk of developing vascular pathology (Figure 2B). Smoking was related to more severe hippocampal atrophy (Figure 2C). Hypertension, overweight, and excessive alcohol consumption were related to a steeper cognitive decline (Figure 2D). Importantly, a higher total number of risk factors was associated with faster cognitive decline, but not with any of the other outcomes.

**Conclusion:**

Common modifiable dementia risk factors differentially affect intermediate brain and cognitive outcomes. These risk factors seem more strongly linked to cognitive decline than to upstream brain pathologies, however, further replication is needed.